# Media coverage of health issues and how to work more effectively with journalists: a qualitative study

**DOI:** 10.1186/1471-2458-10-535

**Published:** 2010-09-08

**Authors:** Julie Leask, Claire Hooker, Catherine King

**Affiliations:** 1 Senior Research Fellow and Conjoint Senior Lecturer, National Centre for Immunisation Research and Surveillance, The Children's Hospital at Westmead, Discipline of Paediatrics and Child Health, University of Sydney, Australia; 2 Senior Lecturer, Medical Humanities, Centre for Values, Ethics and Law in Medicine, University of Sydney, Australia; 3 Information Manager, National Centre for Immunisation Research and Surveillance, The Children's Hospital at Westmead, Australia

## Abstract

**Background:**

The mass media has enormous potential to influence health-related behaviours and perceptions. Much research has focused on how the media frames health issues. This study sought to explore how journalists in Australia select and shape news on health issues.

**Methods:**

The study involved semi-structured interviews with 16 journalists from major Australian print, radio and television media organisations reporting on avian influenza and pandemic planning. Journalists, including reporters, editors and producers, were interviewed between October 2006 and August 2007. Thematic analysis was used to draw out major lessons for health communicators.

**Results:**

Journalists routinely attempted to balance different, sometimes competing, aims amidst significant operational constraints. They perceived the most trusted sources on health issues to be respected and independent doctors. Specialist health and medical reporters had a more sound technical knowledge, channels to appropriate sources, power within their organisations, and ability to advocate for better quality coverage.

**Conclusions:**

An awareness of how to work with the media is essential for health communicators. This includes understanding journalists' daily routines, being available, providing resources, and building relationships with specialist health reporters.

## Background

It is well recognised that the media plays an enormously influential role in public responses to health issues. The mass media - print, television, radio and internet - has an unparalleled reach as a communication mechanism [1]. It has substantial power in setting agendas, that is, *what *we should be concerned about and take action on, and framing issues, that is, *how *we should think about them [2].

Public health professionals have always been sensitive to the persuasive power of the mass media [3,4]. In fact public health has often had the challenging task of both using the media to influence health practices while countering this same influence where it encourages unhealthy choices. These issues are especially acute in a crisis, such as the current A(H1N1) 'swine' influenza pandemic. On such occasions hitting the right pitch is crucial, and difficult. Health communicators may need to advocate rapidly and effectively for the public adoption of basic preventive measures, like handwashing, while such messages may be displaced in a mass media dominated by discussion of technical interventions, such as thermal scanners.

For public health communicators to attempt to achieve their goals, it is essential to understand how the mass media works. Over the past three decades, a number of works have investigated how news is sought and shaped by journalists within media organisations [5-8]. Others have proposed strategies for increasing news coverage of significant health and medical issues [9]. But these strategies need to be pursued carefully. It is well recognised that the mass media, especially its traditional components, print, television and radio, is in many ways a *poor *vehicle for the communication of scientifically accurate information about health and medicine, prone to sensationalism, sins of omission, and sheer inaccuracy. Many health and medical scholars and professionals would agree with those doctors and news commentators who recently stated that the media fails health services, and that the structural limitations on news production made "evidence based journalism" a "forlorn hope" [10-12].

Significantly though, none of these dispirited commentators had researched the views of on-the-ground newsroom journalists, producers or editors, the people who select, shape and present news [13]. While many studies of news production processes exist, few address implications for public health directly, nor explore ways in which it can extend its reach and impact through the mass media. Some do identify that health communication will always be limited within the mass media [14,15]. The fact that health professionals and journalists have different values and goals - not to mention different concepts of validity, objectivity and significance - is as well known as the frustrations that arise from these differences [14,16]. Journalists tend to use anecdotal or rhetorical rather than statistical evidence; rely on expert testimony rather than on publications; emphasise controversy rather than consensus; and represent issues in terms of polarities rather than complexities [14]. There are significant barriers to increasing quality of health and medical reporting. These include: lack of technical training for journalists [6], the time constraints of news production [7,15], and the commercial imperatives that drive story selection and headlines [6,15].

However, it is not all bad news. Research on reporters' attitudes and practices shows that their concerns and aspirations are often much closer to those of the health and medical professionals they report on, and sometimes place them in some conflict with editors and producers [8,15,17]. Editors and producers in turn confront tensions between economic and structural imperatives and their own sets of values and commitments. This complexity makes researching journalists very important, and suggests the possibility for improved engagement with the mass media [7,18].

Furthermore, public health can have a more productive engagement with the mass media if there is greater understanding of how health news is constructed within media organisations. Armed with this knowledge, public health professionals engaged in advocacy can have the greatest possibility of working with, rather than against, the media.

Accordingly, this study aimed to identify how journalists from all sectors of the news production process worked within their organisations to select, shape and present health news stories. It was conducted as part of a wider study examining the production of news on avian influenza. The study offers recommendations for public health professionals in working more effectively with the media. It adds to the relatively small pool of empirical literature on journalistic practices in health and medical contexts [7,8,19].

## Methods

### Study design

This paper reports on some of the findings from a study that was designed to seek journalists' perceptions and reporting practices on avian/pandemic influenza. We conducted interviews with journalists between October 2006 and August 2007, many months after a peaking of media reporting of the avian influenza threat that occurred in Australian between October and December 2005. The interviews were semi-structured and divided into two parts. Participants were told they would be asked general questions about journalistic practice followed by specific questions about pandemic influenza reporting. This paper focuses on the general questions, including how stories were selected, how the focus or 'angle' was formed, and how journalists perceived ethical, social and other issues in their work.

### Recruitment and sampling

Ethical approval was gained from The University of Sydney Human Research Ethics Committee (Ref 9345). Participants were Australian reporters, editors and producers in newsprint and broadcast (radio and television) media known to have been involved with reporting on avian/pandemic influenza. We recruited to ensure a mix of major cities and type of media organisation including commercial and non-commercial, broadsheet and tabloid. Both specialist medical journalists and non-medical journalists were included. Criterion sampling was used for print journalists. We approached those with nine or more bylines selected from 1200 Australian print media articles on pandemic influenza sourced from the Factiva news media database at the peak of reporting on the avian influenza threat in late 2005. For television and radio participants, advice was sought from two interviewees to name journalists who were likely to report frequently on this topic. We sought more print journalists because television and radio news is often selected from the major daily newspapers and hence they have a more important role in selecting and framing news. Our study was focused on journalists working in the traditional mass media, since this media still tends to be identified as setting the agenda for public discussion, and because those working in traditional mediums were often reporting, or reported, online as well.

### Data collection

The majority of interviews were conducted via telephone by one researcher (JL) and lasted approximately 30 minutes. Interview confidentiality was explained to each participant along with a description of how their quotes would be attributed in reports in terms of their general work designation. Consent forms were signed and all interviews were digitally recorded, transcribed verbatim and imported into NVivo v.7 qualitative software.

### Analysis

This study focuses on the general aspects of the journalists' work. Analysis was informed by a priori interests in how media messages are framed along with the question of how health professionals using the media can best work with journalists [9,20]. Coding was primarily undertaken by one researcher (JL) and proceeded iteratively according to the principles of thematic coding [21]. Preliminary codes were identified and then categorised and developed into more abstracted themes that captured participants' contextual responses to the constraints of media production processes. A subset of eight transcripts were also reviewed independently by a second researcher (CK) and themes discussed and revised. The coded text was reviewed by all researchers and re-categorised to demonstrate connections and relationships between themes in a process loosely influenced by recent evolutions in grounded theory traditions [22]. Each theme was then reviewed by all authors to discuss general findings, exceptions and differences between participant groups.

## Results

Twenty-three journalists were approached. Three declined; three did not respond; and one interview recording failed. Sixteen interviews were analysed. The journalists were senior with a median of 14.5 years in journalism (range: 5-37 years). Most had worked for multiple organisations and in differing roles although they usually stuck to the one medium, for example, print. Of the entire sample, seven were specialist health/medical reporters. Table 1 shows the type of media and role for the participants.

**Table 1:** Interviewed Australian journalists in study of news reportage of health issues.

	Reporter	Producer/Editor	*Total*
Newspaper	6	2	*8*

Radio	2	1	*3*

Television	2	3	*5*

*TOTAL*	*10*	*6*	*16*

Our primary finding was that journalists routinely attempted to balance different, sometimes competing, aims (e.g. between depth and newsworthy quality) amid significant operational constraints. In this paper, we identify these constraints through an examination of journalists' daily newsgathering routines. We show how journalists were committed to various ethical principles within these constraints. Finally, we argue that specialist medical reporters have much greater capacity to produce and advocate for quality health stories. While this analysis focuses on the general aspects of news reporting, the journalists made their responses in the context of a discussion on reporting on avian influenza, then a significant (if fading) health risk issue.

### Daily newsgathering routines

#### Time: opportunities, limits, deadlines

The daily schedule and strict deadlines dominated the days of all the journalists in this study. Most journalists had only a concentrated time window between about 10 am and 2 pm to assemble their story: to gather background information, become familiar with technical aspects of the issue, find and conduct interviews, update editors or producers, and check the accuracy of drafts or scripts. The stories themselves were usually brief, particularly in broadcast news, where they ranged between 1.5 and 3.5 minutes. As one journalist commented,

On one axis you have maximum accuracy, integrity, detail - all of those wonderful things. And then on the other one you've got time. Your job is to do the best you can within that parameter. (Newspaper medical reporter)

In both television and print media, the stories for the day would be identified, reviewed and allocated during a mid morning news conference of producers/editors who determined what stories would run and often their angle. A second mid afternoon news conference finalising prominence effectively formed the deadline for new stories, after which all but the most newsworthy stories would be excluded.

#### Selecting news and forming the angle

A very high volume of information inflow meant editors, producers and reporters had to exercise much judgement on selection of news. Some participants had difficulty in clearly articulating what drove them to choose one story over another. One producer described a general ability fostered by many years in journalism.

Our key job is news judgement, which all journos learn about and spend their whole lives developing and changing etc. (Producer, television news)

Other participants reflected on what made a story newsworthy and, while a general question, it is likely their responses suggest they had avian and pandemic influenza in mind. A story's ability to gain entry to the day's news was influenced by it emerging during peak news gathering times; by momentum; sensationalism (fear, death, destruction, such as a threatened pandemic); novelty (new, fresh, exciting, different, quirky such as a new medical technology); whether it involved disagreement or controversy; was something that affected audiences directly; had local relevance; or had a moral element. Sometimes the criteria were medium specific, for example, good visuals for television. These elements of newsworthiness tended to be those common to all news sectors, not just health.

What's new, fresh, exciting, different, what people are going to say, 'Gee, is that right'? (Newspaper medical reporter)

The interviews highlighted the derivative nature of particularly broadcast news, which largely confined itself to expanding on stories emerging from the wires and morning newspapers. All daily news and current affairs journalists reported watching the wire feeds and their competitors in both print and broadcast media. This meant that news selection and angle formation were oriented around distinguishing oneself from one's competitors. For journalists with the ability to source their own stories (usually medical reporters), going local - using local experts, local audiences and hand picked real-life cases - was regarded as the premier means of providing a novel angle and compelling story.

It's quite competitive - you're trying to get some sort of fresh angle that nobody else has, which means you work the contacts quite a bit, depending on which area, you'll be ringing around people who you know might be in the know or have a pretty good idea of what's going on. (Newspaper medical reporter)

#### Finding and quoting sources

Journalists' sources of information for stories were both passively acquired (e.g. media contacts, media releases), particularly in the morning, and actively sought (e.g. calling local medical experts, medical journal contents). The best people to interview for health stories were seen to be accessible, independent, highly respected in their fields, and preferably doctors. All journalists needed access to expert sources who could rapidly respond to requests for background information, interviews or verification and who could condense information into ready-made soundbites. Non-medical reporters with little science background found it important to access brief, readily digestible information, and key point fact sheets were particularly helpful with more technical issues. Cases of interest and angles conveying the direct relevance of the story to audiences were also sought.

Often I do get pointy headed stories and twist them around to a more consumer oriented angle....But if I can't find a line that means something for a suburban family then it's usually going to be difficult to get it into a newspaper. (Newspaper medical reporter)

In broadcast media, the critical resource was the visual image. The availability and vividness of images was a strong determinant of whether a story ran at all, and how prominent it was.

### Managing constraints: the ethics of reporting

Journalists were keenly aware of shortcomings in media stories as a result of constraints on time, space and resources. Many compensated for these constraints by adhering as best they could to their tenets of quality journalism: being informative, responsible and critical.

#### Being informative

As in other studies, journalists articulated an overwhelming commitment to keeping the public informed. This was foundational to their resolution of ethical issues in their professional lives. While they were aware of and concerned about public impacts of some reporting, they felt the *act *of reporting to be of primary importance:

Most journalists would always err on the side of reporting first and that is your first duty and then social impact that follows from that. You don't really gain anything by lying to people and giving them a false sense of security. (Television current affairs producer)

The exceptions to the primacy of informing were in reporting suicides and bomb scares, where not reporting better served the public interest.

#### Not fear mongering

Journalists frequently commented that the commercial goals of the media often sensationalised stories, particularly through the use of dramatic headlines.

Just as a joke, the chief of staff will go 'now I'd like you to create a bit of fear and panic out there, there are some storms coming in'. It's just a joke, but there is an element of truth in it, in that the most sensational part will be your lead. (Radio news reporter)

They countered this tendency through maintaining accuracy and thoroughness in the stories that accompanied these headlines. One radio producer mentioned vocal tone and choice of experts.

It's about just the way you report, I guess it's about speaking in measured tones and going to experts who are not likely to be as emotional and emotive. (Radio producer)

#### Being critical; avoiding others' agendas

Journalists frequently commented that one of their most important roles was to question and critique those on whom they were reporting. As well as being important in itself, this allowed journalists to avoid being seen to be used to further any particular agenda.

The media is not the public relations wing of the health department. We are not there simply to report what they want to tell the public - though we will usually do that also. But our role is to ask challenging, independent questions. (Newspaper medical reporter)

In line with other studies, "objectivity" was much prized by this study's journalists. It was attained, not by evaluating evidence, but rather by quoting respected health experts and by finding and reporting on dissenting opinions.

It's a matter of balance, and again you're casting around in the time you've got - which may not be very much - to ensure that if it is some quite spectacular claim being made, that it's well-based and if there are people who disagree you quote and report what they've got to say about it. (Newspaper medical reporter)

### A key role: the specialist reporter

Specialist health and medical reporters had much greater capacity to produce better quality health stories. This occurred despite the similar seniority of non-specialist reporters and related to key factors specific to their role. Their familiarity with technical aspects of medicine and health enhanced their ability to comprehend and accurately report complex issues. They were able to build networks of contacts among experts with whom they could develop trusting relationships, and so gain greater depth and insight into stories, and pass on tips about 'the talent' - expert sources - to chiefs of staff and junior journalists. Specialist medical reporters enjoyed considerable autonomy within news organisations, and were able to select, pursue and form angles for stories. They were more likely to produce a health story sourced from their own contacts, rather than derive news and expert comments from other news.

Their seniority within their organisation enabled them to negotiate with producers and editors, including advocating for 'worthy' (but not necessarily high rating) stories. Said one newspaper medical reporter, "*A big part of the battle is trying to ensure that it gets a run that you think it's worth*." As arbiters of reporting quality, specialist reporters had a significant gatekeeper role for letting stories in, and *keeping them out*, of the paper.

If all I've done all day long is keep three really crap stories out of the paper then I consider I've done a good day's work. And sometimes that can be quite a lot of work if somebody higher up than me has got themselves all ignited about something. Then there's a lot of work to do to hose people down and to bring these things round. (Newspaper medical reporter)

They also corrected technical inaccuracies of other journalists in their organisation.

A mistake gets repeated again and again and again...so any specialist reporter will put out a national note, saying 'attention all, please ensure this is correct'. (Radio news general reporter)

## Discussion and recommendations

Our study highlighted that time constraints and access to resources and technical expertise remain the major issues for journalists in producing high quality health and medical stories. We also found that the derivative nature of most stories fosters homogeneity in story selection and angle and prevents a degree of critical journalism. The increased use of syndicated material (e.g. Reuters) also erodes localism which, according to Chadwick, "weakens the civic conversation at state, regional, municipal and even neighbourhood level" [23]. The ability of medical reporters in our study to source and localise stories counterbalanced this. It is then significant that the decline of resources for mainstream media is threatening the existence of the specialist reporter.

Our study also found that reporters shared the same concerns as health professionals about the depth, accuracy and social impact of their reporting. For example, they considered this in how they sought to present and portray stories of the pandemic threat. Many would assuage the dissonance felt between the commercial imperatives of media and the need for ethical reporting by articulating their commitment to informing, checking the accuracy of stories, balancing sensational headlines with sober, factual reporting, locating respected expert sources and by remaining 'objective' - aware of, but not actively supporting, any particular agenda, and covering a range of opinions. It appeared that notions of objectivity and facts helped them to rationalise this potential conflict but also prevented them from acknowledging that *all *news is constructed and hence never solely 'factual' [24].

We have described the importance of specialist health and medical reporters: having baseline levels of technical knowledge to help them maximise technical accuracy; negotiate with editors and producers about the selection and angle of medical stories; and build and sustain networks of expert sources. Their gatekeeping role allowed them to include important stories and exclude poor ones, based on their own notions of quality. Thus a journalists' ways of seeing the world and their frameworks would have an important bearing on story selection. The resulting gatekeeping could function to maintain quality insofar as radical, 'inaccurate' messages were often excluded as a result. This served the media's function of getting accurate information 'out there' such as during an emerging pandemic. However, it could also reduce diversity in story, angle and source selection. As Hodgetts and colleagues have argued, it could succeed in privileging dominant biomedical notions of health while failing to represent minority views or those more marginalised discourses such as the social determinants of health [8,19,25]. Indeed, in discussing the pandemic problem and its solutions, we found that medical reporters conceptualised it as a biomedical problem, subject to solutions such as antiviral medication and vaccines rather than public health, social or policy solutions such as social distancing, quarantine, agricultural or trade regulation. This finding was reflected in the media coverage to which these reporters contributed (personal communication, Samantha Siripol, University of Sydney, 2009).

We relied on what journalists told us they did and their perceived roles. In such studies there is always the chance that participants present an idealised account of how things should be rather than what they are. However, the grounding in a real life reporting issue (avian/pandemic influenza) may have helped them locate their accounts in actual practice. The study is limited by a relatively small number of editors and producers not allowing the full exploration of how a particular role or medium influenced news production. Further, the study's core aim was to understand avian influenza reporting. Participants' anticipation of this topic contextualised the responses to the general questions that we report in this paper. In particular, selection of source tended to focus on sources most able to comment on the topic of influenza and less on health stories in general. While not a limitation, it is an important context to these findings.

Our study suggests that improved public health advocacy will result from the following recommendations for public health professionals and others involved with the media, particularly:

1. **Timing**. For soliciting a journalist's interest in an issue, a morning phone call during peak story sourcing time is best. Avoid the late afternoon with newspaper and evening news reporters. Large news organisations may be a more efficient way to broadly distribute stories (e.g. Reuters, AAP).

2. **Be available**. Being readily accessible is necessary for journalists to gain background, interview or film you and to check stories. This means ready telephone contact, timely return of calls, and a willingness to drop other things.

3. **Provide pre-prepared resources**. Depending on the topic, this may include fact sheets, visual aids, and soundbite quotes. Anticipate lack of technical familiarity and provide definitions and distinctions (e.g. an antiviral is not the same as a vaccine).

4. **Find a personal touch**. To make a story compelling to the ordinary citizen, journalists will seek individuals who can provide a personal account of the impact of the health issue on them. Providing an average 'Joe Blow' angle will help the issue get media attention.

5. **Stay networked**. Find and cultivate specialist medical reporters, be willing to provide them with background, and help supply them with stories if available.

6. **Appeal to ethical values**. Journalists prioritise the reporting of information over almost all other considerations. However, they are also sensitive about the potential negative impacts of media coverage of public health issues. It is therefore worth making an explicit appeal to a journalist's values and making a case for covering, or not covering, a particular issue or taking a particular angle (always understanding that the journalist needs to exercise his or her own autonomy and judgment in the end). Senior and specialist reporters have power to advocate for how a story is reported and may be willing to make changes if a suitable rationale is given.

More extensive general guidelines for how doctors [26] and scientists can most effectively communicate with or respond to the mass media have been drawn up by various organisations and are available on the internet (see, for example, http://www.sciencemediacentre.org).

These suggestions focus on media advocacy in a mainstream media context which remains the most influential avenue for public health communicators to target. However, the rise of social media and the decline of the traditional consumption patterns of mainstream news media presents challenges and opportunities in how public health professionals now work with the media. The opportunities include the capacity for public health professionals to have direct and unfiltered input into issues via blogging and Twitter; to take advantage of social networking sites to promote and advocate for health; and the ability for leading health organisations whose integrity and achievements have made them highly trusted to become the authoritative sources of accurate health information, communicated on their own terms. However, in a far more fragmented media context there is the increasing diminution of the role of specialist reporters with resulting loss of baseline technical knowledge, gatekeeping and thoughtful, investigative health journalism.

Regardless of the future media landscape, we argue that public health communicators should be strategic in how they work with journalists, keeping in mind the above and other guidelines for communicating with the mainstream media. In this way we can minimise the faults and maximise the benefits of our current media environment.

## Competing interests

The authors declare that they have no competing interests.

## Authors' contributions

Contributors: JL and CH designed the study. JL conducted most of the interviews. JL and CK developed initial codes. All authors reviewed the codes, developed the themes and wrote the paper. All authors read and approved the final manuscript.

Funding: JL and CK are funded by the National Centre for Immunisation Research and Surveillance (NCIRS) which provided funding for the study. NCIRS is supported by the Australian Government Department of Health and Ageing, the NSW Department of Health and The Children's Hospital at Westmead. CH is funded by the University of Sydney.

This research was undertaken independent of any of its funding sources.

## Pre-publication history

The pre-publication history for this paper can be accessed here:

http://www.biomedcentral.com/1471-2458/10/535/prepub
